# Block of T-type calcium channels by protoxins I and II

**DOI:** 10.1186/1756-6606-7-36

**Published:** 2014-05-09

**Authors:** Chris Bladen, Jawed Hamid, Ivana A Souza, Gerald W Zamponi

**Affiliations:** 1 Department of Physiology & Pharmacology, Hotchkiss Brain Institute, University of Calgary, 3330 Hospital Drive, Calgary, NW, Canada

**Keywords:** Calcium channels, ProTx I, ProTx II, T-type blockers, Electrophysiology

## Abstract

**Background:**

Low-voltage-activated (T-type) calcium channels play a crucial role in a number of physiological processes, including neuronal and cardiac pacemaker activity and nociception. Therefore, finding specific modulators and/or blockers of T-type channels has become an important field of drug discovery. One characteristic of T-type calcium channels is that they share several structural similarities with voltage-gated sodium channels (VGSCs). We therefore hypothesized that binding sites for certain sodium channel blocking peptide toxins may be present in T-type calcium channels.

**Findings:**

The sodium channel blocker ProTx I tonically blocked native and transiently expressed T-type channels in the sub- to low micro molar range with at least a ten-fold selectivity for the T-type calcium channel hCav3.1 over hCav3.3, and more than one hundred fold selectivity over hCav3.2. Using chimeras of hCav3.1 and hCav3.3, we determined that the domain IV region of hCav3.1 is a major determinant of toxin affinity, with a minor contribution from domain II. Further analysis revealed several residues in a highly conserved region between T-type and sodium channels that may correspond to toxin binding sites. Mutagenesis of several of these residues on an individual basis, however, did not alter the blocking effects of the toxin. ProTx II on the other hand preferentially blocked hCav3.2 and significantly shifted the steady state inactivation of this channel.

**Conclusions:**

ProTx I blocks hCav3.1 both selectively and with high affinity. Domain IV appears to play a major role in this selectivity with some contribution from domain II. Given the structural similarities between sodium and T-type calcium channels and the apparent conservation in toxin binding sites, these data could provide insights into the development and synthesis of novel T-type channel antagonists.

## Background

Low-voltage-activated (LVA) or “T-type” calcium channels are encoded by one of three different types of Cav3 α1 subunits (Cav3.1, Cav3.2 and Cav3.3, also known as α1G, α1H, and α1I, respectively) whose membrane topology is similar to those of sodium channels [1]. They are activated by small membrane depolarizations and display rapid activation and inactivation kinetics [2] and they are responsible for triggering low-threshold depolarizations that in turn lead to the initiation of action potentials. Consequently, they are thought to be important for regulating neuronal and cardiac pacemaker activity, and disruption of their normal activity can contribute to cardiac hypertrophy [3-5] and neuronal hyperexcitabilty disorders such as epilepsy and pain [6-9]. Similarly, mutations in Cav3.2 T-type calcium channels have been linked to absence seizures [6,7] and up-regulation of Cav3.2 T-type channel activity in primary afferent fibers has been linked to the development of chronic pain [8,9]. Indeed, depletion of Cav3.2 results in hyposensitivity to pain [10,11].

Modulators and blockers of T-type calcium channels may be useful in elucidating the exact role of these channels in cell signaling pathways and may be exploited for therapeutic purposes. Identification of drugs and molecules that selectively interact with T-type calcium channels has, however, so far proven difficult, although recently novel small organic scaffolds for T-type channel inhibitors have been derived from blockers of other calcium channel subtypes, such as L-type channels [12-14].

Another class of molecules that are known to be effective blockers of voltage gated ion channels are polypeptide toxins. The effects of toxins on ion channels have been extensively documented [for review see Catterall et al., [15] and one toxin isolated from scorpion venom (kurtoxin) is known to be a potent blocker of T-type calcium channels [16]. This toxin however has been shown to also block high-voltage activated calcium channels [17]. More recently, two peptide toxins (ProTx I and ProTx II), isolated from Tarantula venom, have been shown to be potent blockers of both sodium and calcium channels [18-20]. Given the structural similarities between these two classes of ion channels, we tested to what extent these two toxins inhibited T-type calcium channels and identified channel structural determinants of toxin block.

Our results reveal that ProTx II selectively blocks human Cav3.2 (hCav3.2) albeit with far less efficacy than previous reports suggested [18]. ProTx I on the other hand potently and preferentially blocks human Cav3.1 (hCav3.1) in the sub-micro molar range. We therefore focused on ProTx I and used chimeras of hCav3.1 and hCav3.3, as well as sequence alignment between T-type and Nav channels and toxin interaction sites, to determine that Domain IV of hCav3.1 and, to a lesser extent Domain II, are key toxin interaction regions.

## Results and discussion

### ProTx II is a preferential blocker of hCav3.2

**Figure 1:**
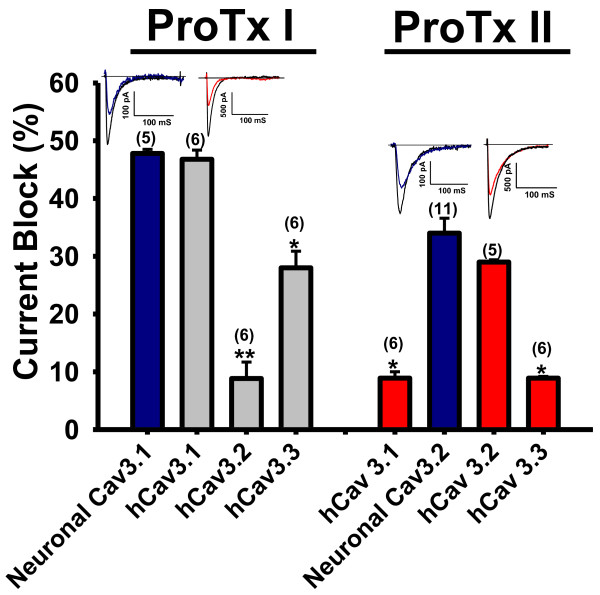
**Tonic block of mouse neuronal or human Cav3.X [T-type] calcium channels induced by either 1 μM application of ProTx I or 1 μM application of ProTx II.** For recordings from native channels, ProTx I was tested on mouse thalamic neurons, ProTx II was tested on mouse DRG neurons, which respectively, expressed Cav3.1 and Cav3.2 channels. Shown are representative current traces from these experiments showing similar ProTx I block of native thalamic mouse T-type currents (first trace) and recombinant human Cav3.1 (second trace), and similar ProTx II block of mouse DRG T-type current (third trace) and recombinant human Cav3.2 (fourth trace) (control traces are depicted in black). Error bars reflect standard errors, asterisks denote statistical significance relative to either hCav3.1 [ProTx I] or hCav3.2 [ProTx II] [*p < 0.05, **p < 0.01]. Currents were elicited by stepping from a holding potential of −110 mV to a test potential of −20 mV.

ProTx II was originally identified as potent inhibitor of sodium channels with fifteen to hundred- fold selectivity for hNav1.7 versus other sodium channels [18,19]. It was also reported to be a potent inhibitor of L-type calcium channels and to mediate a greater degree of inhibition of Cav3.1 versus Cav3.2 [18]. Our observations indicate that ProTx II in fact blocks hCav3.2 more potently than the other T-type calcium channels. The toxin blocked endogenous Cav3.2 channels in acutely isolated mouse DRG neurons [21] with similar potency [Figure 1]. ProTx II also dramatically shifts the steady state inactivation of hCav3.2 towards more hyperpolarized potentials [Table 1A] which produces additional inhibition at typical neuronal resting membrane potentials. Interestingly, although ProTx II only weakly blocked hCav3.1 and hCav3.3, there were significant negative shifts in the half activation potentials of these channels in the presence of as little as 1 μM of the toxin [Table 1A]. Together, these data indicate that ProTx II interacts with hCav3.2 channels in a way similar to β-scorpion toxin interactions with sodium channels [15], and that it also modulates the gating behavior of hCav3.1 and hCav3.3 channels.

**Table 1:** **Summary of biophysical parameters of various T-type calcium channels in the absence or presence of A, 1** μ**M ProTx II and B, 1** μ**M ProTx I**

**A**					
**Clone**	**Current block (%) ProTx II (1 μM)**	**V**_**0.5**_**act (mV) Con**	**V**_**0.5**_**act (mV) ProTx II (1 μM)**	**Vh (mV) Con**	**Vh (mV) ProTx II (1 μM)**
hCav3.1	8.9	-51.6	-64.5**	-73.7	-76.5
hCav3.2	29	-49.4	-52.1	-64.2	-76.5*
hCav3.3	8.9	-50.0	-60.7	-79	-77
**B**					
**Clone**	**Current block (%) ProTxl (1 μM)**	**V**_**0.5**_**act (mV) Con**	**V**_**0.5**_**act (mV) ProTxl (1 μM)**	**Vh (mV) Con**	**Vh (mV) ProTxl (1 μM)**
hCav3.1 wt	50	-52.3	-52.3	-74	-76
hCav3.2 wt	8	-52.6	-58.8*	-64	-75*
hCav3.3 wt	26*	-50.1	-50.1	-79	-75

Asterisks denote statistical significance relative to hCav3.2 [A] or hCav3.1 [B] for current block, or relative to wild type for all other parameters [*p < 0.05, **p < 0.01].

### ProTx I is both a potent and selective blocker of hCav3.1

**Figure 2:**
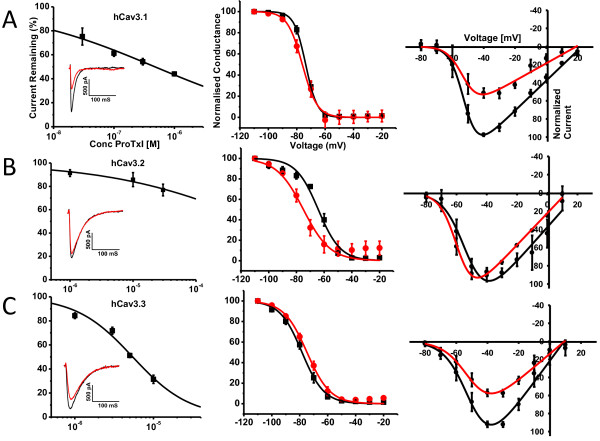
**A,B,C, ProTx I Dose response (left), steady state inactivation (middle) and current voltage relations (right) for hCav3.1, hCav3.2 and hCav3.3, respectively.** Steady state inactivation and current voltage relations were recorded prior and after application of 1 μM ProTx I. Ensemble dose response curves for tonic channel inhibition by ProTx I were fitted via the Hill equation and IC50’s for hCav3.1, 3.2 and 3.3 were 0.64, 94.6 and 5.4 μM respectively. Insets are representative current traces of each calcium channel before and after (red trace) application of 1 μM ProTx I (Note that the trace for hCav3.1 is the same as in figure 1). All other data were fitted with the Boltzmann equation and are from multiple paired experiments [n = 5-6 per channel]. Note the negative shift in both the half activation and steady state inactivation potential in the presence of 1 μM ProTx I for hCav3.2 despite this concentration having minimal effect on tonic block.

ProTx I belongs to the inhibitory cysteine knot (ICK) family of peptide toxins that are known to interact with voltage-gated ion channels [18,22] and to be potent inhibitors of voltage-gated sodium channels [18-20,23]. More recently, several studies demonstrated that ProTx I could also potently block the human Cav3.1 calcium channel [18,24]. One of these studies showed that ProTx I was selective for hCav3.1 over hCav3.2 and that this selectivity may be in part attributed to the S3-S4 linker in Domain IV of hCav3. [24]. In our hands, when applied to transiently expressed hCav3 channels, ProTx I preferentially inhibited hCav3.1 channels, with less block of hCav3.3 and only very little inhibitory effect on hCav3.2. We tested 1 μM ProTx I on endogenous Cav3.1 that comprise a large portion of T-type current in isolated mouse thalamic neurons [25], and observed inhibition that was similar to that of hCav3.1 [Figure 1, Table 1B]. Contrary to previous findings, we did not observe any significant positive shifts in the half activation potential of hCav3.1 in the presence of 1 μM ProTx I in spite of its potent inhibitory effects, nor was the gating of hCav3.3 channels affected [Figure 2A,C and Table 1B]. In contrast, hCav3.2 channels underwent shifts in both the half-activation and inactivation potential in the presence of the toxin [Figure 2B, Table 1B].

### The domain IV region of hCav3.1 is important for ProTx I block and function of hCav3.1

**Figure 3:**
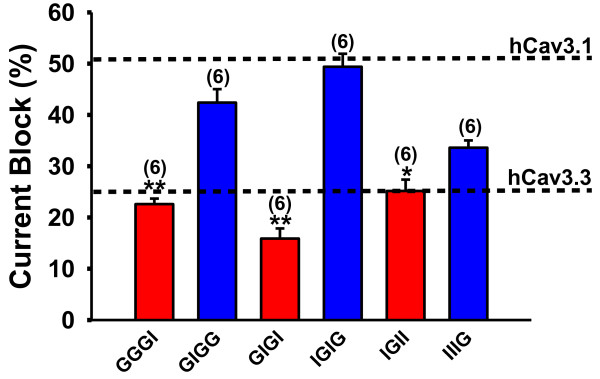
**Tonic block of hCav3.1-hCav3.3chimeras by 1 μM application of ProTx I.** Chimera nomenclature is as follows: hCav3.1 sequence in domains I-IV is denoted by “G”, hCav3.3 sequence is denoted by “I”. Note that block of hCav3.1 and Chimeras that contain Domain IV of hCav3.1 are similar (see top dashed line). The bottom slashed line represents percent tonic block of 1 μM ProTx I of wild type hCav3.3. [n = 5-6 per channel, at 1 μM]. Error bars reflect standard errors, asterisks denote statistical significance relative to hCav3.1 [*p < 0.05, **p < 0.01]. Currents were elicited by stepping from a holding potential of −110 mV to a test potential of −20 mV.

We had previously constructed a series of chimeric channels in which we had swapped various membrane domains between Cav3.1 and Cav3.3 [26]. We used a subset of these chimeras to ascertain which of the membrane domains were responsible for the differences in ProTx I blocking effects on these two channel subtypes. As shown in Figure 3, chimeras that contained the Domain IV region of hCav3.1 exhibited a degree of block that was similar in magnitude to that of the wild type channel, whereas constructs that contained Cav3.3 sequence in this domain behaved liked the wild type Cav3.3 channels [Figure 3]. These data indicate that Domain IV is a major determinant of ProTx I action on T-type calcium channels. In addition, replacing domain II in the IGIG chimera with corresponding Cav3.3 sequence (IIIG) weakened the blocking effect, suggesting that domain II may also contribute to toxin action. We also examined the effect of the toxin on the gating behavior of these sets of chimeras. Most of the chimeras did not undergo a toxin induced change in half-activation or inactivation potential as expected from our observation with wild type channels, although two constructs exhibited a minor depolarizing shift in half activation potential when the toxin was applied [Table 2]. We note that a previous study in which ProTx I was shown to induce a dramatic shift in half activation potential of Cav3.1 used a cDNA derived from rat [18]. We therefore tested ProTx I on rat Cav3.1 and although there was a slight positive shift in activation, it did not reach significance. We did, however, observe a small but significant negative shift in the steady state inactivation that was consistent with previous findings [18] and which curiously contrasts with our observation with human Cav3.1 where no significant shift was observed [Table 2].

**Table 2:** **Summary of biophysical parameters of human Cav3.1, rat Cav3.1 and hCav3.1-hCav3.3 chimeras in the absence or presence of 1** μ**M ProTx I**

**Chimera**	**Current block (%) ProTx I (1 μM)**	**V**_**0.5**_**act (mV) Con**	**V**_**0.5**_**act (mV) ProTx I (1 μM)**	**Vh (mV) Con**	**Vh (mV) ProTx I (1 μM)**
hCav3.1 wt	50	-52.3	-52.3	-74	-76
GGGI	23**	-43.5	-44.5	-75.3	-77.5
GIGG	43	-56.9	-49.0 #	-76.1	-75.9
GIGI	16**	-41.1	-33.5 #	-73.2	-73.2
IGIG	50	-41.8	-34.6 #	-68.9	-69.3
IGII	25*	-42.5	-41.0	-74.0	-73.7
lIIG	35	-41.7	-38.8	-72.5	-73.7
rCav3.1 wt	60	-57.6	-54.2	-75.1	-80.9 #

Asterisks denote statistical significance relative to hCav3.1 for current block. The hash tag denotes significance relative to control [#, *p < 0.05, **p < 0.01].

### Substitution of individual amino acid residues in the putative toxin blocking sites do not affect ProTx I block of hCav3.1

**Figure 4:**
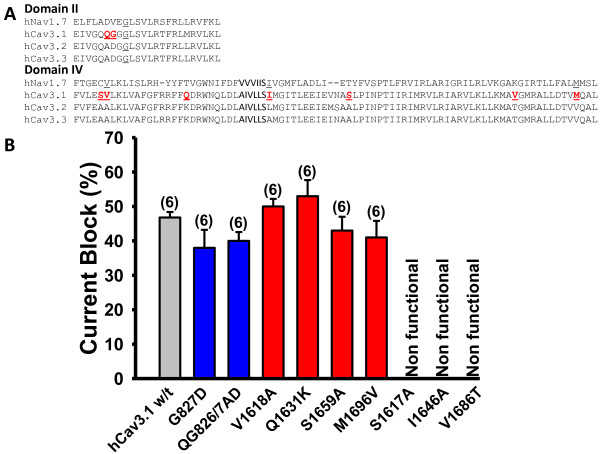
**A Sequence alignment of the human sodium channel 1.7 (hNav1.7) versus the three T-type calcium channels (sites targeted with mutagenesis highlighted in red). B**, Tonic block of human Cav3.1 and Cav3.1 mutants induced by 1 μM application of ProTx I. Domain II mutations are denoted in blue, Domain IV mutations are shown in red [n = 5-6 per channel, at 1 μM]. Note that the last three mutations in Domain IV produced a non-functional channel (NF). Error bars reflect standard errors. Currents were elicited by stepping from a holding potential of −110 mV to a test potential of −20 mV.

Given the high degree of homology between T-type calcium channels, we used ClustalW2 multiple sequence alignment of both the Domain II and Domain IV regions of these channels to determine if there were any sites that were unique to hCav3.1 that might be involved in toxin block, taking into consideration the loci of amino acid residues that are known to be involved in toxin block of voltage-gated sodium channels [27-29]. Our alignment results (Figure 4A) yielded nine candidate residues, two in Domain II of hCav3.1 and seven in Domain IV. To determine whether these residues may be involved in ProTx I block of hCav3.1, we replaced all nine residues with corresponding residues in Cav3.3, and then assessed ProTx I block of these mutant channels.

As shown in Figure 4B and Table 3, three of the amino acid residues resulted in non-functional channels. Surface biotinylation experiments revealed that these mutant channels were trafficked appropriately to the cell surface (data not shown), indicating that their inability to support whole cell currents was not due to an absence of expression. Instead, since these substitutions are in the region thought to be involved in inactivation, it is likely that these substitutions disrupted this mechanism. Of the remaining mutations, none resulted in a significant decrease in ProTx I block. Two of the mutations V1618A and Q1631K both displayed a significant negative shift in their half inactivation potential compared to wild type channels. In addition, the V1618A mutant also showed a significant positive shift in half activation potential compared to wild type [Table 3A]. These data indicate that although individual candidate amino acid residues cannot account for the blocking effects of this toxin, these residues do play a minor role in regulating the gating behavior of the channel.

**Table 3:** **A, Summary of biophysical parameters of hCav3.1 and hCav3.1 domain IV mutants and B, Summary of biophysical parameters of hCav3.1 domain II mutants in the absence or presence of 1** μ**M ProTx I**

**A**					
**DOM IV mutant**	**IC50 Tonic ProTx I (μM)**	**V**_**0.5**_**act (mV) Con**	**V**_**0.5**_**act (mV) ProTx I (1**μ**M)**	**Vh(mV) Con**	**Vh (mV) ProTx I (1 μM)**
hCav3.1 wt	0.64	-52.3	-52.3	-74	-76
hCav3.1 V1618A	0.66	-51.5	-46.8	-79*	-77
hCav3.1 Q1631K	0.92	-53.2	-54.3	-84*	-82
hCav3.1 S1659A	1.29	-53.1	-50.8	-77	-79
hCav3.1 M1696V	1.27	-51.5	-49.1	-74	-79
**B**					
**DOM II mutant**	**Current block (%) ProTx l (1 μM)**	**V**_**0.5**_**act (mV) Con**	**V**_**0.5**_**act (mV) ProTx I (1 μM)**	**Vh(mV) Con**	**Vh (mV) ProTx l (1 μM)**
Q826A	40	-53.6	-51.2	-75.2	-73.6
G827D	38	-52.4	-52.7	-77	-72
QG826-7 AD	40	-51	-48.2	-74	-76.3

Asterisks denote statistical significance relative to wild type hCav3.1 [*p < 0.05].

### Comparison with previous work

Previous studies have shown that the tarantula venom peptides ProTx I and ProTx II inhibit voltage-gated sodium channels by shifting their voltage dependence of activation to more positive potentials [18,20]. Our results show that ProTx I preferentially blocked hCav3.1 at sub-micro molar concentrations, but we did not observe any shift in half activation potential. Contrary to previous findings [18], ProTx II appeared to preferentially block hCav3.2. This toxin block caused a significant negative shift in half inactivation voltage of hCav3.2, but in contrast with previous studies on sodium and calcium channels, no significant change in half activation potential [18,30,31]. The apparent differences between some of our results and those of previous studies, may be in part be due to the different expression systems, recording methods and clones used. In previous studies, HEK cells and *Xenopus* oocytes were used to express rat Cav3 channels and toxin effect and channel kinetics were measured on tail currents as an indicator of potency. We attempted to address this discrepancy by using ProTx I on a rat Cav3.1 clone available to us and although our results showed a small positive shift in the voltage-dependence of activation [Table 2], it did not reach significance. Further experiments will need to be conducted to determine the precise biophysical interactions of this toxin with T-type calcium channels, and how toxin actions are affected by different experimental conditions.

## Conclusions

Our data show that ProTx I and ProTx II potently and preferentially block hCav3.1 and hCav3.2 respectively. These two toxins block and modify T-type calcium channels using mechanisms similar to their interaction with sodium channels [18,20]. Their effect on the voltage dependence of inactivation is reminiscent of β-scorpion toxin interactions with sodium channels [15]. Overall, our data suggest that both ProTx I and ProTx II may be useful towards exploring the gating mechanisms of T-type calcium channels. Finally, the apparent similarities in the toxin binding sites between Nav and Cav channels may provide an insight into the synthesis of more potent antagonists that act on either or both of these channel subtypes.

## Materials and methods

### CDNA constructs

Human Cav3.2 cDNA was kindly provided by Dr. Terrance Snutch (University of British Columbia, Vancouver, Canada). Human Cav3.3 was obtained from Dr. Arnaud Monteil (CNRS Montpellier, France), human Cav3.1 was described previously by our laboratory [32] and human Cav3.1 and Cav3.3 chimeras were also described previously [26].

### Chemicals

Unless stated otherwise, chemicals were purchased from Sigma (St. Louis, MO). Both ProTx I and ProTx II were purchased from Alomone Labs (Jerusalem, Israel) and were dissolved in external recording solution at the stock concentration of 1 mM. All subsequent dilutions were also made in external recording solution.

### tsA-201 cell culture and transfection

Human embryonic kidney tsA-201 cells were cultured and transfected using the calcium phosphate method as described previously [33]. Briefly, 6 μg of T-type calcium channel Cav3.1, Cav3.2, and Cav3.3, α1 subunits were transfected together with 0.5 μg Enhanced green fluorescent protein (EGFP) DNA (Clontech) as a marker. Cells were re-suspended with 0.25% (w/v) trypsin-EDTA (Invitrogen) and plated on glass cover slips a minimum of 3 to 4 hours before patching and kept at 37°C and 5% CO2.

### Isolation of neurons

Thalamic neurons were isolated as described previously [34]. Briefly, thalami of adult mice were dissected out, cut into small pieces and then digested in papain (Worthington, LS003126) containing culture media. After digestion, the tissue was washed and triturated for neuron dissociation. Thalamic neurons were then seeded at low density onto coverslips pretreated with poly-d-lysine (Sigma, P7280). Dorsal Root Ganglia (DRG) neurons were isolated as described previously [21]. Briefly, DRG from adult mice were removed and placed in Ca^2+^ and Mg^2+^-free Hank’s Balanced Salt Solution, containing (in mM): 140 NaCl, 5.3 KCl, 0.4 KH_2_PO_4_, 0.3 Na_2_HPO_4_, 6 D-glucose, 10 HEPES, and 2 mg/mL collagenase (Type I, Worthington, Lakewood, New Jersey), and 200 units of DNaseI (Worthington, Lakewood, New Jersey). Ganglia were then incubated for 45 min at 37°C and subsequently placed in media supplemented with 10% fetal bovine serum to stop digestion. Cells were then dispersed with fire polished Pasteur pipettes and plated on glass coverslips coated with 100 μg/mL poly-L-lysine.

### Electrophysiology

Whole-cell voltage-clamp recordings on tsA- 201 cells were performed at room temperature 2 to 3 days after transfection. Whole-cell voltage-clamp recordings on Neuronal cells were performed at room temperature, the following day after isolation. The external recording solution for all calcium channel recordings contained (in mM): 114 CsCl, 20 BaCl_2_, 1 MgCl_2_, 10 HEPES, 10 Glucose, adjusted to pH 7.4 with CsOH. For voltage-clamp recordings on neuronal cells, 5 μM CdCl_2_ was also added to the external solution to inhibit high voltage activated calcium channels. For all recordings, the internal patch pipette solution contained [in mM]: 108 CsMeSO_4_, 2 MgCl_2_, 11 EGTA, 10 HEPES adjusted to pH 7.3 with CsOH. The internal solution was supplemented with 0.6 mM GTP and 2 mM ATP, which were added directly to the internal solution immediately before use. Liquid junction potentials for the above solutions were left uncorrected. Recordings were digitized at 5 kHz and low-pass filtered at 1 kHz.

Toxins were prepared daily in external solution and applied locally to cells with the use of a custom built gravity driven micro-perfusion system that exchanges solution in approximately one second [35]. Currents were elicited from a holding potential of −110 mV and were measured by conventional whole-cell patch clamp using an Axopatch 200B amplifier in combination with Clampex 9.2 software (Molecular Devices, Sunnyvale, CA). After establishment of the whole cell configuration, cellular capacitance was minimized using the analog compensation available on the amplifier. Series resistance was <10 MΩ and was compensated >85% in all experiments. Data were filtered at 1 kHz (8-pole Bessel) and digitized at 10 kHz with a Digidata 1320 interface (Molecular Devices). In addition to collecting the raw data, an online leak-subtraction protocol was used in which four pulses of opposite polarity and one-quarter amplitude were applied immediately before the test protocol. For current–voltage relation studies, the membrane potential was held at −100 mV and cells were depolarized from −80 to 20 mV in 10 mV increments. For steady-state inactivation studies, the membrane potential was depolarized by test pulses to −20 mV after 3.6-s conditioning pre-pulses ranging from −110 to −20 mV. The current amplitude obtained from each test pulse was then normalized to that observed at a holding potential of −110 mV.

### Data analysis and statistics

Data were analyzed using Clampfit 9.2 (Molecular Devices). Preparation of figures and curve fitting was carried out with Origin 7.5 software (Northampton, MA, USA). Current–voltage relationships were fitted with the modified Boltzmann equation: *I =* (*G*_max_*(*V*_m_*-E*_rev_))/(*1 +* exp((V_0.5act_-V_m_)*/k*_a_)), where *V*_m_ is the test potential, *V*_0.5act_ is the half-activation potential, *E*_rev_ is the reversal potential, *G*_max_ is the maximum slope conductance, and *k*_a_ reflects the slope of the activation curve. Data from concentration-dependence studies were fitted with the equation *y* = A2 + (A1-A2)/(1 + ([C]/IC_50_)^P^) where A1 is initial current amplitude and A2 is the current amplitude at saturating drug concentrations, [C] is the drug concentration and *P* is the Hill coefficient. Statistical significance was determined by paired or unpaired Student’s *t*-Tests and one-way or repeated measures ANOVA followed by Tukey’s multiple comparison tests. Significant values were set as indicated in the text and figure legends. All data are given as means +/− standard errors. Steady-state inactivation curves were fitted using the Boltzmann equation: *I* = 1/(1 + exp((*V*_m_-*V*h)/*k*)), where *V*h is the half-inactivation potential and *k* is the slope factor.

### Ethical standards

All experiments performed in this manuscript comply with the laws of Canada.

## Abbreviations

ProTx I and II: Protoxin I and II; VGCC: Voltage gated calcium channel; WT: Wild type; hCav: Human voltage activated calcium channel; Nav: Voltage activated sodium channel; IV: Current voltage; Va: Half activation potential; DRG: Dorsal root ganglion.

## Competing interests

The authors declare that they have no competing interest.

## Authors’ contributions

CB designed and carried out electrophysiology experiments, point mutations and drafted the manuscript. JH designed and created hCav3.1-hCav3.3 chimeric channels. IAS designed and conducted biochemistry experiments. GWZ directed the study and edited the manuscript. All authors read and approved the final manuscript.
